# Outdoor smoking as a nuisance to non-smokers: The case for smoke-free outdoor public spaces in dense urban areas

**DOI:** 10.18332/tpc/145502

**Published:** 2022-02-21

**Authors:** Jeroen Bommelé, Bethany Hipple Walters, Saskia van Dorsselaer, Marc C. Willemsen

**Affiliations:** 1 The Netherlands Expertise Centre for Tobacco Control, Trimbos Institute, Utrecht, The Netherlands; 2 Massachusetts General Hospital, Boston, United States; 3 Department of Health Promotion, Maastricht University, Maastricht, The Netherlands

**Keywords:** smoking, secondhand smoke, health geography, local tobacco control, urban density

## Abstract

**INTRODUCTION:**

Despite the growing number of smoke-free spaces, many non-smokers continue to be involuntarily exposed to secondhand smoke outdoors and on public streets. Both theory and research suggest that people living in densely populated urban areas are more likely to smoke than those living in less densely populated areas. Consequently, non-smokers in densely populated urban areas might be more likely to be exposed and feel annoyed by secondhand smoke outdoors. We investigated whether the extent to which non-smokers feel annoyed by secondhand smoke exposure in outdoor public spaces is related to urban population density.

**METHODS:**

We used cross-sectional survey data from the Netherlands ‘Module Substance Use’ survey (2020 data, n=9375). This is a nationally representative sample of the adult population in the Netherlands. Using logistic regression models, we investigated whether urban population density predicts both smoking and non-smokers’ annoyance to secondhand smoke exposure outdoors.

**RESULTS:**

We found that smoking rates were associated with urban population density. In the Netherlands, people living in extremely population-dense urban areas were more likely to smoke than those living in non-urban areas (AOR=1.59; 95% CI: 1.25–2.02, p<0.001). Feeling annoyed by secondhand smoke outdoors was also associated with urban population density: non-smokers living in extremely population-dense urban areas were more likely to be annoyed than respondents living in non-urban areas (AOR=1.65; 95% CI: 1.34–2.02, p<0.001).

**CONCLUSIONS:**

These cross-sectional data highlight the importance of comprehensive local tobacco control policy programs that include creating smoke-free outdoor public spaces. This need for such smoke-free outdoor public spaces might be particularly strong in densely populated areas.

## INTRODUCTION

Decades of research has shown that exposure to secondhand smoke could have serious health consequences^1^. While long-term exposure to secondhand smoke increases the risk of illness and death^2^, short-term exposure has been associated with health consequences as well. Research conducted on the effect of short-term exposure on lung function revealed that exposure to secondhand smoke for one hour could result in inflammatory reactions in the lungs and in decreased lung function^3^. It is therefore recommended by the WHO and tobacco control scientists that policymakers implement smoke-free policies to protect non-smokers from the harms of exposure to tobacco smoke^4,5^.

Exposure to secondhand smoke and smoking-related particulate matter is not just a health hazard. Non-smokers also consider this exposure to be a nuisance or annoyance^6^. Further, it is likely that exposure to tobacco smoke would be more of an annoyance in crowded and confined spaces, such as terraces, public transport stops, and near building entrances. In those spaces, non-smokers would be exposed to higher levels of secondhand smoke and would have an increased chance of being exposed due to the increased likelihood of being near a person who smokes. Studies conducted in New Zealand and Japan showed that non-smokers are often annoyed by secondhand smoke exposure in both public and private outdoor spaces^7,8^, while research conducted in Switzerland revealed that non-smokers report being exposed to and annoyed by secondhand smoke in restaurants, cafes, and bars^9^.

### Geographical differences in smoking

Research in the field of human geography has shown how characteristics of people’s physical living environment affect smoking rates^10^. In general, smoking rates tend to be higher in economically depressed and disadvantaged neighborhoods that are more urbanized or densely populated^11-13^.

Pearce et al.^10^ developed a model for explaining such geographical differences in tobacco smoking rates. According to their model, these differences can be explained by both place-based practices and place-based policies (‘regulations’). They consider place-based practices as all behaviors, attitudes, and social norms that promote smoking or that promote smoking cessation. Examples of such practices are social cohesion, social practices, and contagion of smoking^10^. Social cohesion refers to the level in which residents in neighborhoods feel connected, trust each other, and participate in the community. Generally, lower levels of cohesion are associated with higher smoking rates^14,15^. Second, social practices are the social norms within a community about smoking. These norms can either promote smoking or smoking cessation^16^. Finally, contagion refers to the fact that people tend to be socially influenced by the people around them. Smoking is spread through social networks and this is one of the reasons why, for example, children of smoking parents are more likely to start smoking than children whose parents do not smoke^17^.

In addition to identifying various place-based practices, Pearce et al.^10^ also distinguished several area-level policies which promote or reduce smoking. Examples of such policies are restricting tobacco retailing, providing smoking cessation support, and creating smoke-free outdoor public spaces. Restricting the number of tobacco retail outlets within areas could help reduce smoking locally, while reducing the number of retail outlets could make it less tempting to start smoking and less difficult to quit smoking^18-20^. Further, providing smoking cessation support locally can reduce smoking in neighborhoods and communities^21^. To be successful, this support should be targeted to and ultimately reach the most vulnerable groups in neighborhoods and communities, such as people with a low income who smoke, people who smoke with low educational level, and those living in very dense urban areas^22^. Finally, creating smoke-free outdoor public spaces could help change smoking norms locally^23-25^. These changed norms would make it easier for adolescents not to start smoking and for adults who smoke to quit smoking^26^.

### Geographical differences in secondhand smoke exposure

While the framework of Pearce et al.^10^ explains geographical differences in smoking, a number of studies suggest that there are also geographical differences in secondhand smoke exposure among non-smokers. Non-smokers living in urban areas are also more likely to be exposed to secondhand smoke outdoors than those living in non-urban areas^27,28^. A study conducted in Spain showed that people who smoke are more visible in crowded dense areas than in less dense areas, particularly near hospitality venues, public transportation stops, and retail venues^27^. In addition, the level of secondhand smoke exposure appears to be high in those dense places^28^. In dense urban areas, public spaces are shared with many more people than in less dense areas. This increased presence of both people who smoke and their tobacco smoke in dense urban areas would make it more likely that non-smokers feel annoyed by this lingering tobacco smoke.

### This study

We investigate to what extent non-smokers feel annoyed by secondhand smoke in outdoor public spaces and whether there are differences in this, between densely populated urban areas and non-urban sparsely populated areas. We analyze non-smokers’ response to the survey item: ‘In which of the following places do you sometimes feel annoyed by someone else's tobacco smoke?’. We hypothesize that the increased presence of both people who smoke and their smoke in urban areas would make it likely that non-smokers in densely populated urban areas are more frequently bothered by secondhand smoke than those in less dense areas. To the best of our knowledge, this question has not been explored in prior studies. This article, which shares the findings of our research on the question of non-smokers’ annoyance with exposure to tobacco smoke, addresses this gap in knowledge.

## METHODS

### Survey design and respondents

We used data from the 2020 Dutch ‘Additional Module Substance Use’ survey of the Lifestyle Monitor consortium^29^. This survey has been used for in-depth analyses of smoking behavior in the Netherlands. Eligible respondents were selected through a random, representative sample from the municipal population registry. Respondents were first approached by mail and asked to participate in the online version of the survey. A selection of those who did not respond to the initial invitation were approached to complete the survey via telephone (CATI) or face-to-face (CAPI). In 2020, a total of 9424 (unweighted, 9375 weighted) respondents aged ≥18 years participated in the survey. The weighted data were representative of the population of the Netherlands. No other inclusion or exclusion criteria were used.

### Demographics

Respondents indicated their sex, education, migration background, smoking status, and municipality. Among respondents aged 18–25 years, we used their highest level of education as a measure for socioeconomic status. Among respondents aged ≥25 years, we used their highest level of completed education. Education was categorized into ‘low’ (elementary school, lower secondary education or lower vocational education), ‘medium’ (intermediate vocational education or higher secondary education), and ‘high’ (higher vocational education or university).

### Annoyed by secondhand smoke outdoors or on the streets

Non-smoking respondents were asked the question: ‘In which of the following places do you sometimes feel annoyed by someone else's tobacco smoke?’. The phrase ‘on the streets’ is often used synonymously with ‘outdoors’ in Dutch. Respondents were able to select up to three places from a list of 15 response options. The most frequently selected option was ‘outdoors or on the streets’. We therefore used this single item for our analysis. We also selected this item as other places are often non-public (e.g. school grounds) or not visited frequently (e.g. entrances of care facilities).

### Urban density

Urban density was based on the number of (residential) addresses within 1 km of the respondent’s residence. We used categories ‘non-urban’ (<500 addresses), ‘slightly urban’ (500–1000 addresses), ‘moderately urban’ (1000–1500 addresses), ‘highly urban’ (1500–2500 addresses), and ‘extremely urban’ (>2500 addresses).

### Statistical analysis

We used SPSS 27 for all analysis. We conducted two logistic regressions to investigate the association between urban density and both smoking and feeling annoyed by secondhand smoke outdoors.

In the first analysis, the dependent variable was smoking status (Reference: never and former smokers) and the independent variable was urban density (Reference: non-urban). We controlled for sex, age, education level, and migration background.

In the second analysis, the dependent variable was feeling annoyed by secondhand smoke outdoors (Reference: not annoyed) and the independent variable was urban density (Reference: non-urban). We controlled for sex, age, education level, migration background, and smoking status. We only included non-smokers in this second analysis, and respondent’s smoking status was therefore either never smoker (Reference) or former smoker.

## RESULTS

Our sample included slightly more women (50.6%) than men. Most respondents were aged ≥55 years (40.5%), and the majority had high level of education (40.7%). Less than a quarter of all respondents had a migration background (23.5%) and nearly 1 in 6 respondents smoked (17.0%). More than half of respondents lived in either highly urban or extremely urban areas (55.6%). Background characteristics are presented in Table 1.

**Table 1:** Sample characteristics of the 2020 Dutch ‘Additional Module Substance Use’ survey (weighted data) (N=9375)

*Characteristics*	*%*	*n*
**Sex**		
Men	49.4	4629
Women	50.6	4746
**Age** (years)		
18–34	27.0	2529
35–54	32.5	3045
≥55	40.5	3801
**Education level**		
Low	22.8	2084
Medium	36.6	3343
High	40.7	3718
**Migration background**		
None/Dutch	76.6	7179
Western	11.0	1028
Non Western	12.5	1168
**Smoking status**		
Smoker	17.0	1593
Former smoker	33.3	3126
Never smoker	49.7	4655
**Urban density**		
Non-urban	7.7	719
Slightly urban	21.4	2002
Moderately urban	15.4	1441
Highly urban	30.3	2844
Extremely urban	25.3	2370

Due to rounding of weighted data, the totals of the smoking and urban density categories do not add up to N=9375. The level of education of 230 respondents was unknown.

### Smoking behavior

Respondents living in dense urban areas were more likely to smoke than respondents in areas with lower density, p-trend<0.001. Those living in extremely urban areas were, for example, more likely to smoke than those living in non-urban areas (AOR=1.59; 95% CI: 1.25–2.02, p<0.001). Table 2 presents the results of this regression analysis in more detail.

**Table 2:** Predictors of smoking (vs former smokers and never smokers) in the Netherlands in 2020 (N=9375)

	*AOR*	*95% CI*	*p*	*Smokers (%)*
**Urban density**				
Non-urban (Ref.)	1			14.6
Slightly urban	0.98	0.77–1.26	0.893	14.3
Moderately urban	1.24	0.96–1.60	0.106	16.3
Highly urban	1.31*	1.03–1.66	0.026	17.3
Extremely urban	1.59*	1.25–2.02	<0.001	19.9
p-trend			<0.001	
**Sex**				
Men (Ref.)	1			20.8
Women	0.56*	0.50–0.62	<0.001	13.3
**Age** (years)				
18–34 (Ref.)	1			22.5
35–54	0.68*	0.60–0.78	<0.001	17.7
≥55	0.40*	0.34–0.46	<0.001	12.8
**Education level**				
Low (Ref.)	1			20.2
Medium	0.78*	0.67–0.90	0.001	20.5
High	0.35*	0.30–0.41	<0.001	12.3
**Migration background**				
None/Dutch (Ref.)	1			16.0
Western	1.19	1.00–1.42	0.052	19.4
Non-Western	1.08	0.91–1.29	0.352	21.2

AOR: adjusted odds ratio. Results from a multivariate logistic regression model with all confounding variables (full model). The percentages on the right are weighted subgroup smoking rates.

* p<0.05.

### Annoyed by secondhand smoke outdoors

Almost half (40%) of non-smokers felt annoyed by secondhand smoke outdoors or on the streets (Table 3). Other places where non-smokers felt annoyed were on café terraces (39%), near entrances of healthcare facilities (13%), and in bars (9%).

**Table 3:** Settings where non-smokers felt annoyed by secondhand smoke in the Netherlands in 2020 (N=7782)

*Settings*	*%*	*n*
Outdoors or on the streets^a^	40	3076
On a café terrace	39	3065
Near the entrance of a care facility	13	991
In a bar	9	707
At work	9	724
On or near a sports field	6	495
In public transport	6	436
At home	5	400
In a restaurant	4	345
On school grounds	3	266
In places where children play	2	188
In the car	2	125
In a sporting club’s canteen	1	112
Other settings	10	743
None of these settings	32	2487

Respondents were allowed to give multiple answers.

a The phrase ‘on the streets’ is often used synonymously with ‘outdoors’ in Dutch.

The logistic regression showed that urban density is significantly associated with feeling annoyed by secondhand smoked outdoors or on the streets, p-trend<0.001. Non-smokers living in extremely dense urban areas were more likely to be annoyed than those living in non-urban areas (AOR=1.65; 95% CI: 1.34–2.02, p<0.001). Former smokers tend to be annoyed more frequently than never smokers (AOR=0.86; 95% CI: 0.77–0.95, p<0.001). Table 4 presents the results of this regression analysis in greater detail.

**Table 4:** Predictors of feeling annoyed by secondhand smoke outdoors in the Netherlands in 2020 (non-smokers only, N=7782)

	*AOR*	*95% CI*	*p*	*Annoyed (%)*
**Urban density**				
Non-urban (Ref.)	1			30.7
Slightly urban	1.10	0.90–1.35	0.351	32.2
Moderately urban	1.44*	1.16–1.79	0.001	39.3
Highly urban	1.60*	1.31–1.95	<0.001	42.4
Extremely urban	1.65*	1.34–2.02	<0.001	45.6
p-trend			<0.001	
**Sex**				
Men (Ref.)	1			36.6
Women	1.30*	1.18–1.43	<0.001	42.1
**Age** (years)				
18–34 (Ref.)	1			48.9
35–54	0.89	0.79–1.01	0.073	43.7
≥55	0.64*	0.56–0.73	<0.001	30.8
**Education level**				
Low (Ref.)	1			26.0
Medium	1.60*	1.39–1.85	<0.001	38.2
High	2.16*	1.88–2.49	<0.001	48.0
**Migration background**				
None/Dutch (Ref.)	1			38.2
Western	0.98	0.84–1.15	0.821	40.9
Non Western	1.18*	1.01–1.38	0.037	47.2
**Smoking status**				
Never smoker (Ref.)	1			43.2
Former smoker	0.86*	0.77–0.95	0.004	34.1

AOR: adjusted odds ratio. Results from a multivariate logistic regression model with all confounding variables (full model). The percentages on the right are weighted subgroup rates of non-smokers feeling annoyed by secondhand smoke outdoors. In this analysis we used the item ‘outdoors or on the streets’ as outcome variable. The phrase ‘on the streets’ is often used synonymously with ‘outdoors’ in Dutch.

* p<0.05.

## DISCUSSION

In line with previous studies^12,13^, we found that urban density is positively associated with smoking rates. In addition, we found that urban density is also positively associated with the percentage of non-smokers feeling annoyed by secondhand smoke outdoors or on the streets. Non-smokers living in highly dense urban areas were much more likely to feel annoyed by secondhand smoke outdoors than non-smokers living in less dense areas. While the link between urban density and smoking rates has been shown across countries before^12^, this is the first study to establish the link between urban density and non-smokers’ annoyance to outdoor secondhand smoke exposure.

One interesting finding is that never smokers appeared to be more frequently annoyed by secondhand smoke exposure than former smokers. This corroborates earlier findings from the Netherlands in which never smokers were more likely to support smoke-free policies, and were also more likely to believe smoke-free policies are important^30^. This suggest that if the portion of never smokers continues to increase in the Netherlands, both annoyance by secondhand smoke exposure outdoors and support for smoke-free policies may increase too in the future.

### Smoke-free outdoor public spaces

These results highlight the need for smoke-free outdoor public spaces in protecting non-smokers from secondhand smoke exposure and from being annoyed by secondhand smoke. In line with the model of Pearce et al.^10^, this need for smoke-free outdoor public spaces might be stronger in dense urban areas than in areas with lower density. In crowded spaces, people who smoke are more visible^27^ and non-smokers are consequently being exposed to high levels of secondhand smoke^28,31^. Perhaps this increased exposure would explain why non-smokers living in urban areas are more likely to be annoyed by secondhand smoke outdoors. The increased exposure to secondhand smoke in dense areas suggests smoke-free public spaces are more needed in dense areas. As the number of people living in urban areas will continue to rise in the future^32^, the need for smoke-free outdoor public spaces might become even stronger in those areas over the coming years.

Studies that collected data in multiple European countries have shown that secondhand smoke exposure is a continent-wide issue. Non-smokers are exposed to secondhand smoke in a variety of outdoor settings, such as school grounds, hospitals grounds, café terraces, beaches, and even children’s playgrounds^33,34^. As the level of secondhand exposure is directly correlated with the strength of local tobacco control policies, these studies show that implementing smoke-free policies could help reducing secondhand smoke exposure to non-smokers. Currently, very few public spaces in the Netherlands are smoke-free by law. Most smoke-free areas, such as sports grounds, hospital grounds, and some terraces, are only subject to voluntary smoke-free policies. School grounds are the only public spaces where smoking is not allowed by law and where offenders could be fined. There are no smoke-free policies on streets in the Netherlands.

Local policymakers can be reluctant to implement smoke-free policies in outdoor public spaces, fearing a lack of support from the public^35^. Fortunately, support for public smoke-free outdoors spaces, such as playgrounds, building entrances, and public transport stops, is generally high and tends to increase after a smoke-free policy has been implemented^24,30^. Non-smokers may also help keep smoke-free spaces smoke-free, as those who are annoyed by secondhand smoke are more likely to support smoke-free policies^6^ and will ask people who smoke not to smoke in smoke-free spaces^36,37^. This is particularly for those non-smokers who feel other non-smokers would do the same and also ask people who smoke not to smoke in smoke-free places^38^. Thus, local governmental policymakers and owners of public outdoor spaces do not need to feel reluctant to create smoke-free outdoor spaces.

### Comprehensive tobacco control policy programs

Although creating smoke-free outdoor public spaces is likely to increase non-smoking norms and reduce non-smokers’ exposure to secondhand smoke in urban areas, implementing such measures in isolation might not be sufficient to tackle tobacco use and exposure in dense urban areas. Even if almost all public spaces became smoke-free, people who smoke would likely continue to find places to smoke; those who smoke would also increasingly feel like outcasts^39^. A tobacco control policy program that focuses on smoke-free areas only might therefore further stigmatize people who smoke. If so, those smokers would feel disengaged and may avoid smoking cessation services. To be effective, a comprehensive area-level tobacco control policy would also contain additional policies, such as strengthening local smoking cessation support services, and reducing the number of tobacco retail outlets^10^. Cessation support services, in particular, could help reduce local smoking rates substantially, especially if they are able to reach those disadvantaged groups who tend to live in dense urban areas^21,22^. Retail licensing policies and systems can help reduce the number of retail outlets near schools and in disadvantaged areas, thereby also reducing geographical inequalities in smoking^40^. Thus, while smoke-free policies are crucial to reducing non-smokers’ exposure to secondhand smoke, they should preferably be part of a larger comprehensive tobacco control policy program.

### Strengths and limitations

A strength of this study is that we were able to use a large sample size to analyze the association between urban density on the one hand, and smoking and feeling annoyed by secondhand smoke exposure on the other. The weighted dataset was representative of the adult population in the Netherlands. As a result, the findings of this study are generalizable to the larger adult population in the Netherlands.

A limitation of this study might be that it was conducted in the Netherlands, which is the second most densely populated country of all 27 EU member states^41^; 55% of respondents lived in very or extremely urban areas and 8% lived in non-urban areas. The results of this study might be different from those that could be found in non-Western, less urban, and less dense countries. Conversely, the location of our data collection might be a strength of this study. As the number of people who live in urban areas continues to rise, the results found in the Netherlands today may prove helpful in the future to countries that have growing urban areas.

A second limitation of this study is that the data collection was conducted during the COVID-19 pandemic. Although the overall smoking prevalence in this study was lower in 2020 than in a similar study on smoking in the Netherlands (17% vs 20%)^42^, the pandemic was unlikely to have influenced the results of this study, as the focus of this work was on associations and not on prevalence rates. In additional (unreported) analyses, we investigated the robustness of the associations found between urban density and both smoking and annoyance to secondhand smoke outdoors; these analyses showed that both associations existed previously in the 2016 and 2018 data. To this end, we believe the COVID-19 pandemic has not influenced the results of this study.

A final limitation of this study is that we have not been able to measure actual exposure to secondhand smoke among non-smokers. We assumed that the higher smoking rates in dense areas cause increased secondhand smoke exposure among non-smokers, which in turn make non-smokers feel more annoyed about this secondhand smoke. It would have been helpful to show that non-smokers in dense urban areas are indeed exposed more often, but, unfortunately, outdoor secondhand smoke exposure had not been measured in our survey.

### Future research

We have been able to test two of the implications of the model of Pearce et al.^10^ for linking place and smoking. In line with this model, we have shown that urban density is linked to both smoking and the need for smoke-free outdoor public spaces. Future research might also investigate other assumed associations of this model. As the government of the Netherlands recently proposed a nationwide ban on the sale of tobacco in supermarkets and in convenience stores^43,44^, one might, for example, want to investigate the effects of reducing the number of tobacco retail outlets nationally. Such a ban might have a different impact in urban areas than in non-urban areas^18^.

## CONCLUSIONS

This study showed that, in the Netherlands, smoking rates are higher in dense urban areas than in less dense rural areas. It also showed that that urban density is also positively associated with the percentage of non-smokers feeling annoyed by secondhand smoke outdoors. Non-smokers living in highly dense urban areas are much more likely to feel annoyed by secondhand smoke outdoors than non-smokers living in less dense areas. These findings highlight the importance of comprehensive local tobacco control policy programs that include creating smoke-free outdoor public spaces.

## Data Availability

Data sharing is not applicable to this article as no new data were created.
